# Visible Light-Driven Phenol Degradation via Advanced Oxidation Processes with Ferrous Oxalate Obtained from Black Sands: A Kinetics Study

**DOI:** 10.3390/molecules30092059

**Published:** 2025-05-06

**Authors:** Salomé Galeas, Víctor H. Guerrero, Patricia I. Pontón, Vincent Goetz

**Affiliations:** 1 Doctoral School Energy and Environment, University of Perpignan Via Domitia (UPVD), 52 Avenue Paul Alduy, 66100 Perpignan, France; salome.galeas@epn.edu.ec; 2 PROMES-CNRS UPR 8521, PROcesses Materials and Solar Energy, Rambla de la Thermodynamique, 66100 Perpignan, France; 3 Department of Materials, Escuela Politécnica Nacional, Ladrón de Guevara E11-253, Quito 170525, Ecuador; victor.guerrero@epn.edu.ec (V.H.G.); patricia.ponton@epn.edu.ec (P.I.P.)

**Keywords:** α-FOD, low-cost precursor, Fenton like, heterogeneous photocatalysis, organic micropollutants

## Abstract

Ferrous oxalate dihydrate (α-FOD) was synthesized from Ecuadorian black sands for phenol removal from aqueous solutions. Visible light-driven photodegradation kinetics were studied by varying the initial pollutant concentration, solution pH, and α-FOD dosage and by adding peroxydisulfate (PDS), including quenching tests. A representative model of phenol photodegradation was obtained by the Langmuir–Hinshelwood mechanism over a large range of concentrations (apparent kinetic constant, k = 0.524 h^−1^). Almost complete removal was reached within 1 h under dark + 9 h under visible irradiation. The degradation rate was slightly affected by pH in the range of 3 to 9, with a significant improvement at pH 11 (k = 1.41-fold higher). The optimal α-FOD dosage was ~0.5 g/L. Two regimes were observed when using PDS: first, a heterogeneous Fenton-like process during the first few minutes after PDS addition; second, pure photocatalysis to completely remove the phenol. When comparing the two systems, without and with PDS, the half-life time for pure photocatalysis was 2.5 h (after the lamp was switched on). When adding PDS (1.0 mM), the half-life time was reduced to a few minutes (5 min after PDS addition, phenol removal was 66%). The photocatalyst presented remarkable degradation efficiency up to five repeated cycles.

## 1. Introduction

Toxicity and persistence of organic micropollutants in water bodies have been a public concern over the previous decades. Consequently, there is a growing need to develop technologies capable of removing these contaminants from water and reducing their associated risks to human health and the environment. In this context, advanced oxidation processes (AOP) have proven to be effective in the removal of organic pollutants due to the in situ formation of highly reactive radicals (OH•, O_2_•^–^, HO_2_•, SO_4_•^–^, ROO•), which can react with toxic organic species to transform them into smaller and less harmful intermediate molecules, ideally leading to complete mineralization [1]. It is particularly relevant and meets sustainability requirements when solar energy is used to activate these oxidation mechanisms [1,2].

The goal of AOPs is to mineralize organic contaminants, ultimately producing CO_2_, H_2_O, and inorganic ions. However, full mineralization of pollutants can be challenging; therefore, the association of degradation mechanisms constitutes a promising alternative. Hence, the combination of heterogeneous photocatalysis and Fenton-like processes, both of which are categorized as AOPs, has shown great potential for this application [3]. In heterogeneous photocatalytic processes, a semiconductor absorbs a specific type of light irradiation, generating reactive radicals in situ, which promote chemical reactions to degrade organic compounds into less toxic intermediates. TiO_2_ is considered the photocatalyst par excellence. However, it is active only under ultraviolet light (UV, wavelength < 400 nm). Considering that UV radiation only represents ~5% of the solar spectrum, while visible radiation corresponds to approximately 47% [4], using photocatalysts with activity in the visible region constitutes a more feasible, safe, low-cost, and easy-to-operate option. Thus, several visible-active photocatalysts have been developed.

Doping of TiO_2_ nanoparticles with non-metals (N, C, and S), transition metal cations, lanthanides, and noble metals has increased the response of this material in the visible region. Other examples of photocatalysts with activity in this region of the spectrum are CdS, CdSe, InP, GaZnON, WO_3_, Ag_2_O, Cu_2_O, BiVO_4_, Bi_2_MoO_6_, Bi_2_WO_6_, RbPb_2_Nb_3_O_10_, etc. [5]. However, their synthesis often requires high purity, costly reagents, and complex synthesis processes. Therefore, a current trend is to synthesize photocatalysts with activity in the visible region from low-cost precursors (like highly available mineral resources) and use environmentally friendly synthesis routes. Some materials that have attracted special attention are metal–organic frameworks (MOFs). MOFs consist of metal ions or clusters and organic ligands disposed in a way that allows them to have high surface areas, high chemical stability, and the capacity to be functionalized. These interesting features make them suitable for photocatalytic applications [6]. Ferrous oxalate is a coordination polymer with an MOF-like structure that can be found in nature as the mineral humboldtine. This material is photocatalytically active in the visible range. Studies regarding the degradation of dyes and pharmaceuticals are reported in the literature [7,8,9].

On the other hand, Fenton and Fenton-like processes involve the use of a primary oxidant and a catalyst capable of activating it to produce highly reactive species. One of the Fenton-like technologies that has gained attention from the academic and industrial sectors is the sulfate radical-based advanced oxidation processes (SR-AOPs), which consist of the production of sulfate radicals (SO_4_•^–^) to degrade recalcitrant pollutants [10]. SO_4_•^–^ radicals can be produced by the activation of primary oxidants such as peroxymonosulfate (PMS) or peroxydisulfate (PDS). PDS offers advantages, including good solubility in water, stability at ambient temperature, high oxidation potential, and lower cost than PMS [11]. For that reason, its use is spreading widely.

In our previous work [12], Ecuadorian ferrotitaniferous sands were employed as a low-cost precursor to produce α-ferrous oxalate dihydrate (α-FOD). The elaboration route implemented was an eco-friendly hydrothermal method that is deeply studied using oxalic acid and subcritical water. Different operating conditions were screened, i.e., oxalic acid concentration, time duration, and temperature of the hydrothermal treatment applied with or without co-solvent. Following this previous paper, which focused on the elaboration route, this work addresses a deeper kinetic study of phenol degradation via AOPs by using α-ferrous oxalate dihydrate obtained from Ecuadorian black sands. The influence of the AOP’s experimental conditions, such as the pollutant initial concentration, the photocatalyst dosage, and the solution pH on the degradation rate of phenol, is discussed. The effect of the addition of PDS as a primary oxidant was explored as well. The reactive species responsible for the degradation of phenol were identified for the photocatalytic system with and without PDS. Finally, the performance of the synthesized α-ferrous oxalate dihydrate was subjected to five consecutive photocatalytic cycles.

## 2. Results and Discussion

### 2.1. Characterization of the α-Ferrous Oxalate Dihydrate

**Figure 1:**
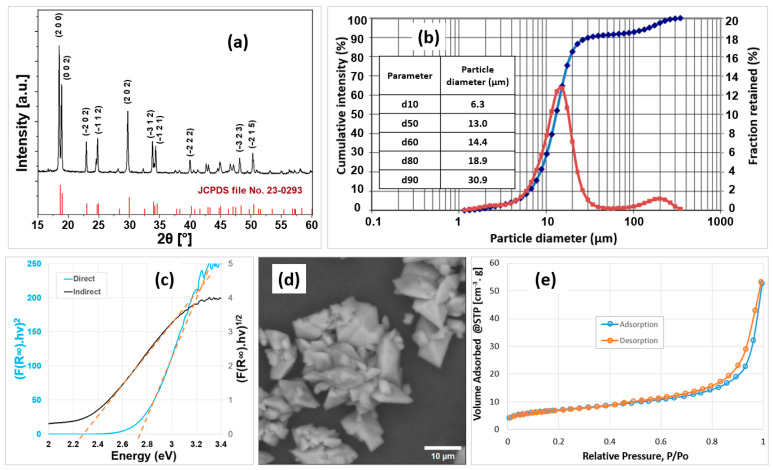
Characterization of the photocatalyst: (**a**) XRPD pattern, (**b**) laser granulometry (Inset: d parameters), (**c**) diffuse reflectance spectrum used for bandgap estimation. (**d**) SEM image, (**e**) N_2_ adsorption–desorption isotherm.

The photocatalyst was characterized to determine its crystalline phase, particle size distribution, bandgap, morphology, and pore characteristics through XRPD, laser granulometry, diffuse reflectance spectroscopy, SEM, and N_2_ adsorption–desorption (BET method and BJH pore size distribution analysis), respectively. The results are displayed in Figure 1 and Table 1.

The XRPD pattern of the as-synthesized photocatalyst is shown in Figure 1a. The main peaks are indexed and compared with the reference data from the JCPDS card No. 23-0293, confirming the formation of single-phase monoclinic ferrous oxalate dihydrate (α-FOD).

The particle size distribution of the α-FOD is displayed in Figure 1b. A bimodal distribution is observed, with over 90% of the particles corresponding to the primary range of 1 to 30.9 µm and a mode size of ~15 µm, where the mode size is the maximum of the frequency distribution [13]. A secondary distribution appears in the curve, with a maximum particle size of ~350 µm. Based on SEM analyses conducted on multiple areas of different samples, no individual particles of this size were observed. Instead, the presence of larger agglomerates composed of smaller primary particles was consistently detected. Furthermore, it is important to note that no grinding step was applied during sample preparation; the material was only dried and sieved. These observations strongly suggest that the secondary peak is the result of particle agglomeration rather than unground large particles. This interpretation is also consistent with the SEM images presented in Figure 1d and Figure S1, where no particles larger than 30 µm are evidenced. Additionally, the SEM micrographs of the as-synthesized α-FOD powder show octahedral-shaped particles. This morphology was reported by other authors for the synthesis of ferrous oxalate from reagent-grade iron salts [14] and from iron-bearing minerals treated with oxalic acid naturally produced by the fungus *Aspergillus niger* [15].

Figure 1e displays the nitrogen adsorption–desorption isotherm. The curve shows a type IV isotherm, according to the IUPAC classification, characteristic of hierarchical mesoporous structured solids (pore diameter between 2 and 50 nm). The surface area calculated by the BET method was 25.13 m^2^/g with a 0.082 cm^3^/g pore volume and an average pore radius of 6.5 nm. These values are comparable to the ones reported by Dhal et al. [16], in which ferrous oxalate presented a BET-specific surface area of 27.55 m^2^/g, a pore volume of 0.086 cm^3^/g, and an average pore diameter of 7 nm.

The bandgap of the α-FOD was estimated by applying Tauc’s method on the solid-state UV-vis spectrum shown in Figure S2. Both direct and indirect band gap estimations were performed [17]. The direct band gap, calculated from the linear portion of the Tauc plot, was found to be 2.78 eV, corresponding to a wavelength of 446 nm. For the indirect transition, a band gap of 2.28 eV was obtained, which corresponds to a wavelength of 544 nm, according to the Planck–Einstein equation [18]. These results are consistent with the literature, where various authors have reported direct transitions with band gap values in the range of 1.90 to 2.75 eV [9,19,20,21], while others have suggested indirect transitions, obtaining bandgap values from 1.91 to 2.4 eV [7,22,23]. The band gap values obtained suggest that the α-FOD is suitable for photocatalytic applications under visible light irradiation, as confirmed by previous studies [12,20,24].

It is noteworthy to mention that two previous studies have determined the positions of the α-FOD conduction (CB) and valence (VB) edge potentials [7,23]. The conduction band in both studies was 0.18 V relative to NHE (normal hydrogen electrode), while the valence edge potentials were calculated at 2.11 V [23] and 2.35 V [7] vs. NHE, respectively. According to these CB and VB edge positions, it can be deduced that reactive oxygen species such as hydroxyl radicals could be photogenerated, considering that the redox potential of the pair OH^–^/OH• is equal to 1.99 V. On the other hand, other species, such as superoxide radicals, would not be produced, as the redox potential of the pair O_2_/O_2_•^–^ is equal to –0.16 V [7,25].

### 2.2. Adsorption, Photocatalysis, and Photolysis

**Figure 2:**
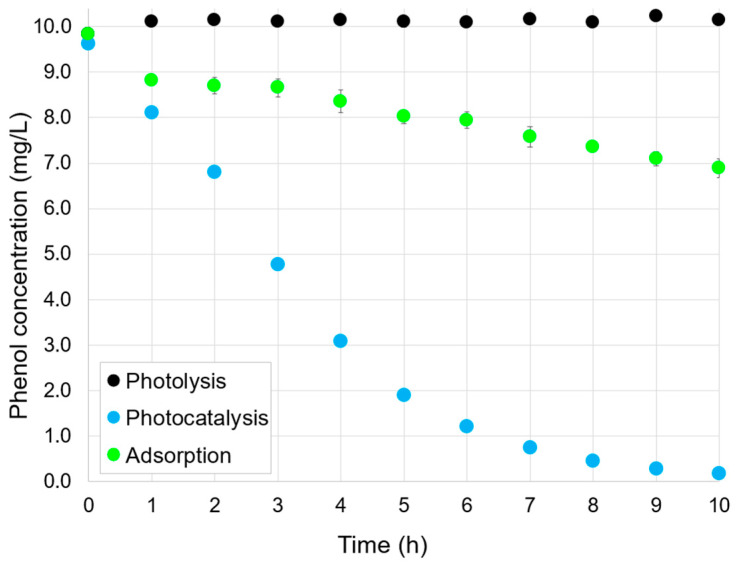
Phenol removal profiles for photolysis, adsorption, and photocatalysis. Experimental conditions: phenol initial concentration, 10 mg/L; FOD dosage, 1 g/L (except for photolysis experiment); experimental time, 10 h under visible light irradiation (photolysis), 10 h in dark conditions (adsorption), and 1 h under dark conditions + 9 h under visible light irradiation (photocatalysis).

Figure 2 shows the concentration profiles of the phenol solution exposed to visible irradiation without photocatalyst (photolysis), photocatalyst without irradiation (adsorption), and photocatalyst under visible irradiation (photocatalysis). As expected, the exposition of the solution to visible light irradiation in the absence of the photocatalyst (black dots) did not have any effect on the phenol removal. On the other hand, pure adsorption (green dots) led to the removal of ~30% of the phenol after 10 h in dark conditions.

In the photocatalysis experiment (blue dots), an initial 1-h dark phase was included before light irradiation to allow partial adsorption of phenol onto the photocatalyst surface. This approach is commonly used in photocatalysis studies to provide a consistent and reproducible starting point for evaluating photocatalytic reactions. During this dark phase, phenol removal reached ~15.8%, indicating that adsorption had begun but had not reached equilibrium. It is important to note that achieving adsorption–desorption equilibrium was not the objective, and the dark period was implemented to simulate realistic conditions for photocatalytic testing.

Following visible light irradiation for 9 additional hours, phenol removal increased significantly, reaching ~98.2%. These results clearly demonstrate the photocatalytic activity of α-FOD under visible light.

It is often difficult to compare the performance of the photocatalysts found in the literature because of the diverse experimental conditions used by the different researchers. However, to give an idea of the photocatalytic potential of the α-FOD obtained in this work, Table 2 examines some studies that use visible light-active photocatalysts for phenol degradation.

As shown in Table 2, a significant performance is observed for the α-FOD, comparable to or even lower than those used in other studies. This, added to the innocuousness of the material and the fact that it was produced from a low-cost precursor and using a generally recognized as safe (GRAS) organic acid (oxalic acid), makes α-FOD an attractive alternative over the expensive and less safe visible active photocatalysts reported herein.

### 2.3. Effect of the Phenol Initial Concentration

**Figure 3:**
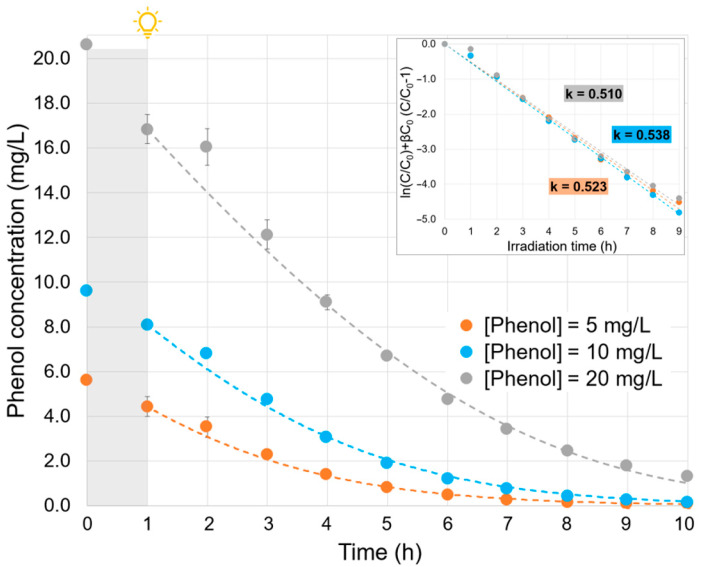
Effect of phenol initial concentration (inset: ln(C/C_0_) + βC_0_ (C/C_0_ – 1) vs. irradiation time plot, with β = 0.12 L/mg and the apparent kinetic coefficient calculation. Experimental conditions: phenol initial concentration, ~5, 10, and 20 mg/L; FOD dosage, 1 g/L; experiment time, 1 h under dark conditions + 9 h under visible light irradiation, neutral pH. Dashed lines represent the calculated profiles with β = 0.12 L/mg and k = 0.524 h^−1^.

The concentration profiles of three phenol solutions with different initial concentrations are depicted in Figure 3. To study the influence of the initial phenol concentration, a Langmuir–Hinshelwood-type formalism was employed to model the kinetics. This model is typically used to describe heterogeneous photocatalytic processes and can be expressed by Equation (1).

(1) $$r=-\frac{dC}{dt}=\frac{kC}{1+\beta C}$$

where *r* (mg/L·h) is the reaction rate, k (h^–1^) is the apparent kinetic constant, β (L/mg) is the adsorption equilibrium constant, C (mg/L) is the pollutant concentration at time t, and t (h) is the irradiation time.

Once integrated, the Langmuir-–Hinshelwood kinetic model leads to a linear relation between ln(C/C_0_) + β C_0_ (C/C_0_ − 1) and the irradiation time, where the slope corresponds to the constant k. To be representative, this formalism must lead to two parameters, k and β, being constant whatever the initial concentrations are, i.e., a good linear regression with the same slope (k value) for the three experiments. This double constraint is respected, at best, with a value of β identified at 0.12 (inset in Figure 3). In this case, the mean R^2^ is higher than 0.99, and the k constants are very close to each other. The average of the three kinetic constants was k = 0.524 h^−1^ and was used to simulate the concentration profiles represented by the dashed lines (Figure 3). Considering the very good agreement between simulated and experimental concentration profiles, the Langmuir–Hinshelwood formalism for the kinetic law was used in the study.

### 2.4. Effect of the Solution pH

**Figure 4:**
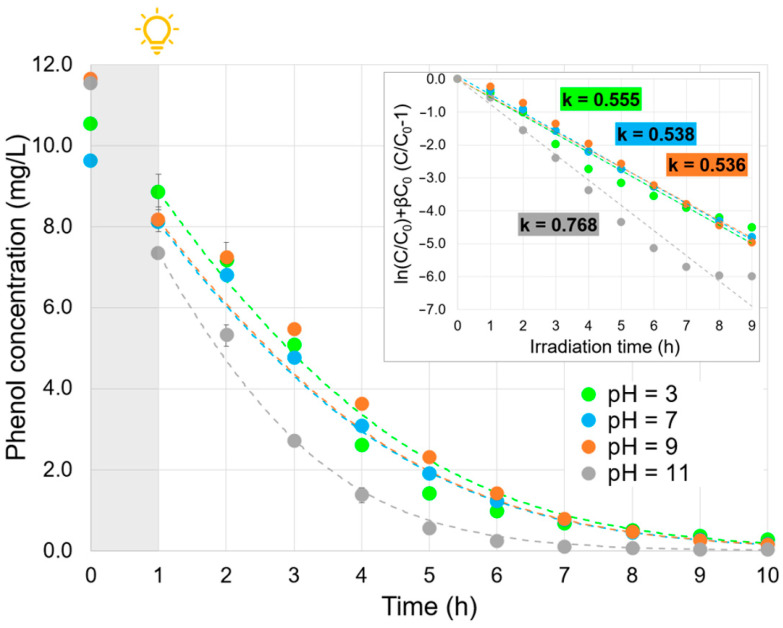
Effect of the solution pH (inset: ln(C/C_0_) + βC_0_ (C/C_0_–1) vs. irradiation time plot, with the apparent kinetic coefficient calculation). Experimental conditions: phenol initial concentration, ~10 mg/L; FOD dosage, 1 g/L; experiment time, 1 h under dark conditions + 9 h under visible light irradiation; pH, 3, 7, 9, and 11. Dashed lines represent the calculated profiles.

Figure 4 illustrates the influence of the initial solution pH on the efficiency of phenol removal. It is well known that the pH of the solution affects the photocatalyst surface charge [30]. The point of zero charge (PZC) is defined as the pH at which the solid surface has an equal number of positive and negative charges and is characteristic of each semiconductor. In this work, the PZC of α-FOD was estimated to be 4.87 using the classical pH drift method (Figure S3) [31], which, although commonly employed for a wide range of materials, including metal oxides and inorganic solids [32,33,34], may present limitations when applied to transition metal-based compounds due to potential surface alterations, especially under strongly acidic conditions.

As shown in Figure 4, adsorption was favored, as the pH was further from the PZC. For instance, at an initial pH of 3 and 7, the adsorption within the first hour under dark conditions was 16%. On the other hand, when the initial pH was 9, the adsorption increased to 30%, and at pH 11, it rose to 36%.

Notably, under alkaline conditions (pH 11), both the surface of α-FOD and phenol, which is predominantly in its deprotonated phenolate form (pKa ≈ 9.89) [35], are expected to be negatively charged, which would typically reduce adsorption due to electrostatic repulsion. However, it is important to recognize that adsorption is not determined solely by surface charge; other factors, such as surface charge density and the size of the adsorbate, also play important roles [36,37].

In this context, the observed increase in both adsorption and photocatalytic efficiency suggests that additional mechanisms are contributing. One likely factor is the higher reactivity of phenolate ions toward hydroxyl radicals (•OH), which could enhance degradation rates [38]. Furthermore, at high pH, the formation of iron–ligand complexes (e.g., Fe–phenolate species) may be favored [39,40], potentially affecting the observed degradation kinetics. It is important to note that phenol concentration quantification was carried out using HPLC. Therefore, the apparent reduction in phenol concentration at pH 11 may not be due solely to adsorption or photodegradation but could also result from the formation of such complexes, which are not detectable by the current HPLC setup.

Accordingly, as observed in the inset of Figure 4, the apparent kinetic constants at pH 3, 7, and 9 were similar (average of 0.543 h^–1^). Conversely, at pH 11, the degradation rate increased, with the kinetic constant k being 1.41-fold the average. It is important to note that even if pH 11 offers the best photocatalytic efficiency, this pH may be difficult to achieve when treating real wastewaters.

### 2.5. Effect of the Photocatalyst Dosage

**Figure 5:**
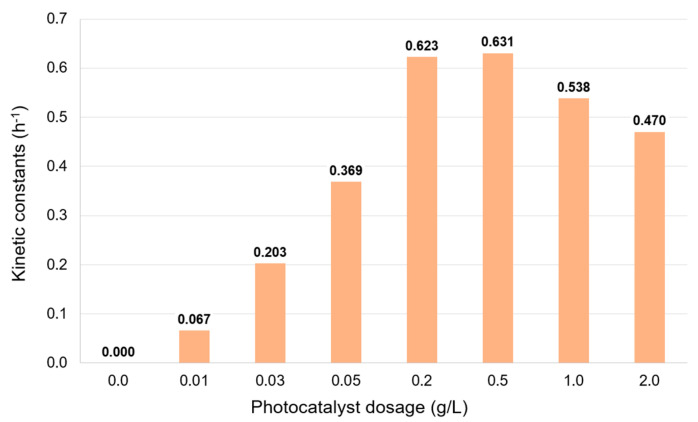
Effect of the photocatalyst dosage on the kinetic constants. Experimental conditions: phenol initial concentration, ~10 mg/L; FOD dosage, 0 to 2.0 g/L; experiment time, 1 h under dark conditions + 9 h under visible light irradiation; neutral pH.

The photocatalyst dosage has a significant influence on the degradation of phenol. The concentration profiles are presented in Figure S4, and the calculated apparent kinetic coefficients for each dosage are displayed in Figure 5. At low dosages, the degradation rate is slow, and it increases with the addition of photocatalyst up to the optimal dosage, around 0.2 and 0.5 g/L. For higher α-FOD concentrations, the degradation rate decreases. This can be explained qualitatively because the photocatalyst suspension uses the incident light impacting the surface of the reactor (for this case, the Erlenmeyer). At low dosages, the photocatalyst is not able to consume all the available light inside the suspension, and some of the light goes through the suspension, being transmitted through the outer wall of the reactor, and therefore not being able to be fully used by the photocatalyst. As the α-FOD dosage increases, the suspension absorbs more and more light and uses it more efficiently until reaching the optimal value of the optical thickness, which, basically, depends on the reactor’s geometry and the photocatalyst concentration [41,42]. From Figure 5, the dosage at which this optimal value of optical thickness is reached is around 0.5 g/L. Hence, if the α-FOD concentration in the suspension exceeds this value, the opacity of the suspension increases, and a dark zone is generated without degradation of phenol by photocatalysis, therefore decreasing the efficiency of the photocatalytic reaction [43]. It is important to note that for the lower α-FOD dosages, the low degradation rates are also influenced by the limited active sites on the contact surface of the photocatalyst, which are able to produce the reactive species for the photocatalytic process [44].

### 2.6. Effect of the Addition of PDS

**Figure 6:**
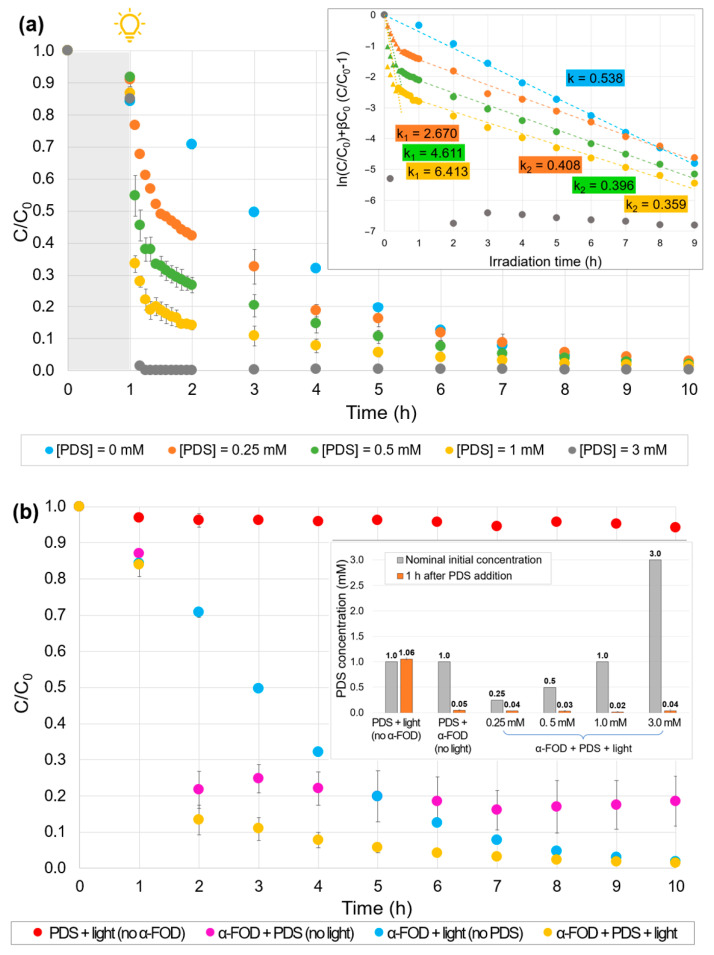
Effect of the addition of PDS: (**a**) ternary systems at different PDS concentrations added (inset: ln(C/C_0_) + βC_0_ (C/C_0_–1) vs. time plot, apparent kinetic coefficient calculation for the two regimes), (**b**) comparison between binary and ternary systems with PDS (inset: PDS concentration measured 1 h after addition). Experimental conditions: phenol initial concentration, ~10 mg/L; FOD dosage, 1.0 g/L; experiment time, 1 h under dark conditions + 9 h under visible light irradiation; neutral pH; PDS concentration, 0 to 3.0 mM.

Figure 6a contrasts the results of phenol degradation during pure heterogeneous photocatalysis and the case in which a primary oxidant is added to the photocatalytic system. PDS was incorporated right after the first hour of adsorption–desorption equilibrium time (1 h in the dark) at concentrations from 0.25 to 3.0 mM. Two different kinetic regimes are evidenced in the inset of the figure (represented by apparent kinetic constants k_1_ and k_2_), except for the curve of PDS = 3 mM, in which the degradation was almost complete within the first few minutes. For the first regime (~30 min after PDS addition), a big difference in the slopes of the curves is evident (k_1_ values), and the higher the PDS concentration, the higher the degradation rate. Hence, when PDS was added in a concentration of 0.25 mM, the time necessary to reduce the phenol concentration to half was around 45 min. If the PDS concentration increases to 0.5 mM, the half-life time is reduced to 10 min, while for 1.0 and 3.0 mM, after 10 min, the degradation of phenol is around 72 and 99%, respectively. It is evident that these results are superior to the half-life time obtained for pure photocatalysis (~2.5 h after the irradiation source is turned on). Moreover, the fast phenol degradation suggests that PDS is consumed during the first minutes of the Fenton-like reaction. Interestingly, during the second kinetics regime, the 0.25, 0.5, and 1.0 mM PDS curves have almost the same slope, corresponding to similar degradation rates (k_2_ values), not so far from the kinetic constant of pure photocatalysis.

Figure 6b compares the concentration profiles of the pure heterogeneous photocatalysis (blue curve) with different catalytic systems. The red curve demonstrates that visible light is not able to activate PDS, as in the case of UV irradiation, which produces an energy of excitation high enough to break the peroxide bond to generate SO_4_^•–^ radicals [45]. The system with photocatalyst and PDS (1.0 mM) in the dark, represented by the magenta curve, shows that α-FOD acts as a Fenton-like activator of PDS. This reaction takes place during the first hour, reaching a phenol removal of 78.4%. Once all the PDS is consumed, the phenol concentration remains constant through the next 8 h of experiment. Similarly, for the ternary system (PDS + α-FOD + light), represented by the yellow curve, a Fenton-like process occurs within the first hour after the addition of PDS with a slight contribution of the heterogeneous photocatalysis by the effect of the light irradiation (phenol removal reaches 86.7%). However, in this case, once all the PDS is consumed, pure heterogeneous photocatalysis continues to occur, and phenol concentration decreases to zero.

PDS concentration was measured 1 h after its addition to the different systems and depicted in the inset in Figure 6b (orange bars). As expected, for all the systems involving α-FOD and PDS (with or without light irradiation), PDS concentration within 1 h is near zero. In contrast, the system PDS/light (without photocatalyst) revealed a PDS concentration of ~1.0 mM unchanged after 1 h. These results corroborate the assumption that all the PDS is consumed by a Fenton-like mechanism during the first hour, while no effect of visible light on the activation of PDS is observed.

To further investigate the possible contribution of homogeneous pathways in the PDS-added system, iron leaching from α-FOD was quantified using atomic absorption spectroscopy (Perkin Elmer Aanalyst 300 atomic absorption spectrometer). After 1 h under dark conditions, Fe leaching was relatively low (~1.4%). However, during photocatalysis (1 h dark + 9 h visible light), leaching increased to 6.4%, and in the presence of PDS under the same conditions, reaching 17.7%. These results indicate that while the solid photocatalyst retains most of its structure, the addition of PDS promotes the partial dissolution of iron into the solution. This suggests that both heterogeneous and homogeneous processes may be contributing to the degradation of phenol, especially in the system with PDS.

### 2.7. Quenching Tests

**Figure 7:**
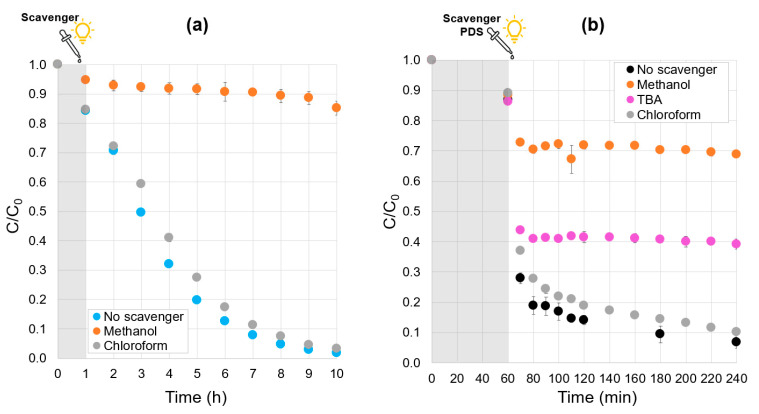
Scavenger quenching tests: (**a**) heterogeneous photocatalytic system, (**b**) addition of PDS photocatalytic system. Experimental conditions: phenol initial concentration, ~10 mg/L; α-FOD dosage, 1.0 g/L; neutral pH; PDS concentration, 1.0 mM. Experiment time: 1 h under dark conditions + 9 h under visible light irradiation for heterogeneous photocatalysis and 1h under dark conditions + 3 h under visible light irradiation for PDS addition system.

The reactive species generated by the systems with and without PDS were investigated through radical quenching tests. The results of pure heterogeneous photocatalysis quenching tests are shown in Figure 7a and demonstrate that methanol was able to scavenge hydroxyl radicals (orange curves), which proved to be the main reactive species. In contrast, superoxide radicals had a negligible effect on the degradation kinetics of phenol, as evidenced by the experiment with chloroform (gray curve). For the test with PDS under visible light irradiation, an additional scavenger was employed to differentiate the effect of the sulfate and the hydroxyl radicals, and the results are presented in Figure 7b. It is known from the literature that two reactive species are principally generated when PDS is activated: hydroxyl (OH•) and sulfate (SO_4_•^–^) radicals [46,47]. Due to the difference in the reactivity towards the different radical species, tert-butanol (TBA) can be used for scavenging OH• radicals (k_OH•_ = 5.2 × 10^8^ M^−1^s^−1^, k_SO4•-_ = 8.4 × 10^5^ M^−1^s^−1^) and methanol to scavenge both SO_4_•^–^ and OH• radicals (k_OH•_ = 1 × 10^9^ M^−1^s^−1^, k_SO4•-_ = 1 × 10^7^ M^−1^s^−1^) [48].

Figure 7b reveals that during the first hour of adsorption under dark conditions, the phenol removal is around 12.4% for all the curves. Within the first 10 min after the addition of the PDS, the system without scavenger reaches a removal of 72.0%, and the phenol concentration continues decreasing until achieving 93.3% phenol removal after 3 h of experiment. For the system with TBA, after the first 10 min, the phenol removal is 56.3%. Advanced oxidation, provided by sulfate radicals not scavenged by TBA, remains very efficient. Alternatively, during the second step, when the PDS concentration is close to zero, the heterogeneous photocatalytic process, which happens mainly thanks to OH• radicals, is almost not observable. Within the next 3 h, a 60.8% removal was reached. Conversely, when methanol is used, the inhibition is greater; hence, for the first 10 min, a 27.2% removal is achieved, and after 3 h, 31.2%. There is a substantial difference between the inhibition effects of both scavengers, suggesting the presence of both SO_4_•^–^ and OH• radicals in the reaction system. It is important to note that the PDS and the scavengers were added almost simultaneously; therefore, the activation of PDS by the α-FOD is fast, which is why there is a rapid drop in the phenol concentration even when SO_4_•^–^ scavenger is added (see orange curve of Figure 7b, when C/C_0_ falls from 0.88 to 0.73). When only OH• radicals’ scavenger is added, the SO_4_•^–^ radical is active and able to degrade phenol; therefore, the concentration drop is very steep (C/C_0_ falls from 0.88 to 0.44 in the magenta curve). These results demonstrate that there is a much larger effect of the SO_4_•^–^ radical during the first step of the reaction, supporting the assumption made previously. In the same manner as for pure heterogeneous photocatalysis, in the presence of chloroform, the second phase of phenol concentration profiles after the PDS consumption confirms the very poor influence of the superoxide radicals; gray and yellow curves are similar.

As demonstrated before, in the case of pure photocatalysis, the phenol degradation by α-FOD is mainly conducted by the action of hydroxyl radicals. Based on the literature, water is the source for obtaining hydroxyl radicals, which are generated by the action of photons on the surface of the photocatalyst in the aqueous suspension. The following chemical reactions represented by Equations (2) to (9) have been proposed for the generation of hydroxyl radicals [49]. Considering the quenching tests in the present work, it is probably Equations (8) and (9) that are mainly involved in the first regime of the hydroxyl radical’s generation.

(2) $$Semiconductor+hv\to h_{VB}^{+}+e_{CB}^{-}$$

(3) $$h_{VB}^{+}+e_{CB}^{-}\to energy$$

(4) $$e_{CB}^{-}+O_{2,ad}\to {O_{2}}^{•-}$$

(5) $${O_{2}}^{•-}+H^{+}\to {{HO}_{2}}^{•}$$

(6) $${{2HO}_{2}}^{•}\to H_{2}O_{2}+O_{2}$$

(7) $$H_{2}O_{2}+e^{-}\to {OH}^{•}+{OH}^{-}$$

(8) $$h_{VB}^{+}+H_{2}O\to {OH}^{•}+H^{+}$$

(9) $$h_{VB}^{+}+{OH}_{ads}\to {OH}^{•}$$

On the other hand, for the photocatalytic system involving the addition of PDS, the degradation of phenol during the first minutes is conducted mainly by the action of the sulfate radicals. It is known that iron-based photocatalysts, such as hematite, are able to react with PDS due to the octahedral sites containing Fe^2+^ to produce sulfate radicals [50]. As for hematite, nearly the same mechanism is expected for α-FOD. For instance, the catalytic reactions in the heterogeneous Fenton-like process happen both in the α-FOD surface and in the solution. Several reactions can take place in this process. At first, the PDS is adsorbed by the Fe^2+^ sites on the photocatalyst surface and reacts with it, producing Fe^3+^, SO_4_•^–^, and SO_4_^2–^ (Equation (10)). Fe^3+^ also reacts with S_2_O_8_^2–^ and produces Fe^2+^ and S_2_O_8_•^–^ (Equation (11)). A cycle between Fe^2+^ and Fe^3+^ occurs on the α-FOD surface as well as in the solution due to the leaking of Fe^2+^ and Fe^3+^. It is important to note that the SO_4_•^–^ radical can also be consumed by itself (Equation (12)) or react with excess Fe^2+^ (Equation (13)) or PDS (Equation (14)). In addition, at a slow rate, SO_4_•^–^ radical can also react with H_2_O to produce OH• radical (Equation (15)), which is able to oxidize the pollutants as well. If these reactions (Equations (12) to (14)) are favored, the efficiency of sulfate radical for the degradation of the pollutant will be hindered. Therefore, the reaction parameters, such as the pH and photocatalyst dosage, will play an important role.

(10) $${S_{2}O_{8}}^{2-}+{Fe}^{2+}\to {Fe}^{3+}+{SO_{4}}^{•-}+{SO_{4}}^{2-}$$

(11) $${S_{2}O_{8}}^{2-}+{Fe}^{3+}\to {Fe}^{2+}+{S_{2}O_{8}}^{•-}$$

(12) $${SO_{4}}^{•-}+{SO_{4}}^{•-}\to 2{SO_{4}}^{2-}$$

(13) $${SO_{4}}^{•-}+{Fe}^{2+}\to {Fe}^{3+}+{SO_{4}}^{2-}$$

(14) $${SO_{4}}^{•-}+{S_{2}O_{8}}^{2-}\to {S_{2}O_{8}}^{•-}+{SO_{4}}^{2-}$$

(15) $${SO_{4}}^{•-}+{Fe}^{3+}\to {Fe}^{2+}+{SO_{4}}^{2-}$$

### 2.8. Photocatalytic Cycling Tests

**Figure 8:**
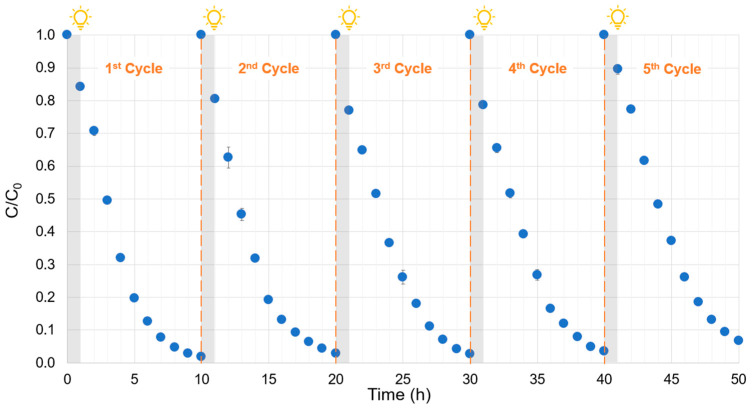
Photocatalytic cycling tests.

The as-synthesized α-FOD demonstrated high degradation performance, which was maintained over five repeated cycles with partial photocatalyst replenishment. As observed in Figure 8, the performance of the photocatalyst was notable during all the cycling experiments, with a low decrease in its activity. For instance, in the first cycle, the removal reached 98%; for the second, third, and fourth cycles, the removal decreased to 97%; and for the fifth cycle, it reduced to 93%. Similar results were reported by other authors: for instance, Liu et al. [51] employed α-FOD microspheres that removed 100% of rhodamine B under visible light irradiation in the first cycle, and for the fifth cycle, the photocatalyst maintained a good performance by degrading around 92% of the pollutant. Fan et al. [7] reported a decrease in the photocatalytic activity of α-FOD on the degradation of rhodamine B from 90% in the first cycle to 82% in the third cycle.

## 3. Materials and Methods

### 3.1. Materials

Ferrotitaniferous black mineral sand with an estimated composition of 0.6FeTiO_3_·0.4Fe_2_O_3_ [52] was used as the precursor for photocatalyst production. Oxalic acid dihydrate (99.0%) was acquired from DQI S.A., phenol (>99.5%), tert-butanol (ACS reagent, ≥99%), and potassium persulfate (PDS, ACS reagent, ≥99%) from Sigma-Aldrich, chloroform (AR) from Loba Chemie, methanol (HPLC grade) from Fisher Chemical, sodium hydroxide (≥99%) and sodium chloride (ACS reagent grade) from Merck, hydrochloric acid (37%, ACS reagent grade) from Pharmco-Aaper, and potassium iodide (reagent grade) from Supelco. Ultrapure water with a specific resistance of 18.2 MΩ-cm, obtained from a purification system (Aquelix 5, Merck-Millipore), was utilized for the preparation of all the aqueous solutions. Nitrogen gas (purity 99.9%) from Linde was employed for the degassing and pressurizing of the hydrothermal reactor.

### 3.2. Synthesis of the Photocatalyst

α-FOD was produced using the method reported in our previous study with slight modifications [12]. In brief, 3 g of ferrotitaniferous black mineral sands were combined with 300 g of a 1.5 M oxalic acid solution and placed in a 500 mL Teflon-lined vessel in the reactor system Berghof BR-500 (Baden-Württemberg, Germany). The system was degassed with N_2_ and pressurized up to 18 bar. The reaction was conducted at 135 °C for 12 h at 350 rpm. Once the reaction was finished, the system was let to cool down, then it was depressurized, and the yellowish product was washed several times with pure water until neutral pH, oven dried overnight at 80 °C, and stored for further characterization and phenol degradation tests.

### 3.3. Characterization of the Photocatalyst

The crystalline phase of the photocatalyst was determined by X-ray powder diffraction (XRPD) on a Bruker D2 Phaser X-ray diffractometer with CuK α radiation (λ = 1.54184 Å) using a 0.02° step size and 0.250 s/step with a LYNXEYE XE-T detector (1D-mode). Scanning electron microscopy (Aspex Corporation, PSEM Express, Delmont, PA, USA) was employed to study the surface morphology and N_2_ adsorption–desorption (Quantachrome Instruments Novatouch LX-1, Boynton Beach, Florida, USA) to determine the specific surface area. The bandgap was estimated using the Tauc method with the absorption coefficient calculated by the Kubelka–Munk function from diffuse reflectance data on a Perkin Elmer UV/VIS Lambda 365 UV–Vis spectrometer. Particle size distribution was determined by laser granulometry in a Horiba LA-950V2 laser scattering analyzer.

### 3.4. Point of Zero Charge

The pH at the point of zero was estimated by employing the classical pH drift method reported by Bhavsar et al. with modifications [53]. While this method was originally developed for carbon-based materials, it has since been widely applied to various inorganic solids and metal oxides, including TiO_2_, hematite, zirconia, and ceramics. It is important to note that this method may present specific limitations when applied to transition metal-based materials like ferrous oxalate dihydrate (α-FOD) due to potential chemical alterations under acidic conditions. Despite these considerations, the method was employed here as a preliminary estimation to gain initial insight into the surface charge behavior of α-FOD in aqueous environments.

Briefly, 50 mL of 0.1 M NaCl solutions were prepared, and their pH was adjusted by the addition of 1.0 M hydrochloric acid (HCl) and sodium hydroxide (NaOH) solutions to reach 2, 4, 6, 8, and 10. These values were designated as the initial pH (pH_0_). These solutions were then transferred to sealed glass containers, and 0.05 g of photocatalyst sample was added to each. The suspensions were magnetically stirred for 48 h at room temperature to allow equilibrium. It is acknowledged that extended equilibration times may increase the possibility of surface transformations, especially at low pH; however, this duration was selected to ensure system stability.

After equilibration, the final pH was measured (pH_f_). These values were used to calculate ΔpH (pH_0_ minus pH_f_) and plotted against pH_0_. The pH at which ΔpH is equal to 0 is the point of zero charge. Experiments were conducted in duplicate to ensure reproducibility, and all the pH measurements were performed using a Jenway 3510 pH meter.

### 3.5. Phenol Removal Tests

All the removal tests were conducted in duplicate in an orbital shaker (TECNAL, TE-1400) at 150 rpm, equipped with an 18 W LED lamp (Sylvania, LED panel SQ 18WDL) with a spectral range in the visible region (emission wavelength from 413 to 750 nm, approx.), and a light intensity of approximately 49.3 mW/cm^2^. In general, for the photocatalytic tests, a determined amount of photocatalyst was dispersed in 50 mL of phenol solution in a transparent borosilicate 125 mL Erlenmeyer using ultrasonication for 1 min (GT SONIC, QTD10). Prior to light irradiation, all photocatalytic experiments were preceded by a 1-h dark phase to allow partial adsorption of phenol onto the photocatalyst. This duration was chosen based on common practice in photocatalysis studies and was intended to establish consistent starting conditions. After this 1-h dark period, the light was turned on for 9 h. The phenol concentration was monitored each hour by high-performance liquid chromatography on an Agilent HPLC L1120 with an Agilent Zorbax Eclipse Plus C18 column (4.6 × 150 mm, 5 µm particle size). Duplicate HPLC injections were carried out. The flow chart of the phenol removal experimental procedure is presented in Figure S5.

#### 3.5.1. Adsorption, Photolysis, and Photocatalysis

The central point conditions for the photocatalyst tests were set at 10 mg/L of phenol initial concentration with 1 g/L of photocatalyst dosage at neutral pH. Adsorption tests for 10 h in dark conditions and photolysis tests under irradiation without the photocatalyst were also conducted for comparison.

#### 3.5.2. Effects of the Initial Phenol Concentration, Solution pH, and Photocatalyst Dosage

The influence of the different parameters was studied. For this, 3 initial phenol concentrations (5, 10, and 20 mg/L), 4 initial solution pH (3, 7, 9, and 11), and 7 photocatalyst dosages (0.01, 0.03, 0.05, 0.2, 0.5, 1.0, and 2.0 g/L) were examined. The pH of the solutions was adjusted by using 1.0 M HCl or NaOH solutions.

#### 3.5.3. Effect of the Addition of PDS

The experiments were performed as follows: By using the central point conditions (phenol = 10 mg/L, photocatalyst = 1 g/L, pH = 7), the PDS was added after the first hour under dark conditions, and the irradiation source was turned on. Different concentrations of PDS were used: 0.25, 0.5, 1.0, and 3.0 mM. Phenol concentration was monitored each hour during the 10 h of experiment. Additional tests were conducted to examine the phenol concentration every 5 min during the first hour after the addition of PDS. Tests with PDS binary systems (photocatalyst + PDS with no light and PDS + light with no photocatalyst) were performed for comparison. PDS concentration after 1 h of addition was measured using the method proposed by Liang et al. [54]. Briefly, 4 mL of the sample containing PDS was mixed with 1 mL of a 100 g/L potassium iodide solution, and its adsorption spectrum was analyzed in a quartz cell at λ = 395 nm (UV-Vis spectrophotometer, Horiba, Duetta, Vermillion, SD, USA).

#### 3.5.4. Quenching Tests

Radical quenching experiments were conducted to identify the possible reactive radicals produced during the photocatalytic degradation of phenol with and without the addition of PDS. For this, methanol and chloroform were used as scavengers of hydroxyl and superoxide radicals, respectively. For the PDS/photocatalyst system, tert-butanol (TBA) was employed as an additional scavenger to differentiate between the effects of sulfate and hydroxyl radicals. The tests were conducted in the same way as the phenol removal tests (detailed in Section 3.5), with the difference that the scavengers were added after the first hour of the experiment when the irradiation source was off. Immediately after the addition of the scavengers (more than 100 times the phenol concentration [55]), the light was turned on, and aliquots of the solution were withdrawn periodically to monitor the phenol concentration by HPLC.

#### 3.5.5. Photocatalytic Cycling Tests

Five cycles of 10 h each were performed to test the photocatalytic efficiency of the α-FOD after repeated cycling (first hour under dark conditions followed by 9 h of visible irradiation). After each cycle, the photocatalyst was separated from the suspension by filtration and washed with pure water, then oven-dried at 80 °C. Later, the recovered photocatalyst was weighed, and the lost amount (~20% of the initial weight) was refilled (with the photocatalyst, which was subjected to the same photocatalytic testing conditions) to obtain the correct amount for each next batch of the recycling test.

## 4. Conclusions

Monoclinic ferrous oxalate dihydrate was synthesized from ferrotitaniferous black sand as a low-cost precursor and used as a visible light-driven photocatalyst for phenol degradation. The characterization revealed that the as-synthesized photocatalyst exhibited octahedral morphology, micrometric particle size (d80 = 18.9 µm), narrow bandgap (2.78 eV for direct transition and 2.28 eV for indirect transition), and a specific surface area of 25.13 m^2^/g. Phenol degradation was explored by varying the initial concentration of the pollutant, the solution pH, and the photocatalyst dosage. The results showed that the phenol degradation phenomenon can be represented by the Langmuir–Hinshelwood model in a large range of phenol concentrations, with an apparent kinetic constant of 0.524 h^−1^. The kinetics of phenol degradation were not influenced by the pH in the range of 3 to 9 but showed an improvement at pH 11 (a pH far enough from the PZC). As expected, the photocatalyst dosage influenced the kinetics of degradation, with the optimal being 0.5 g/L. Additionally, the efficiency of the photocatalyst was evaluated, and α-FOD demonstrated high degradation performance, which was maintained over five repeated cycles with partial photocatalyst replenishment and showed almost no loss in photocatalytic activity.

The effect of the addition of PDS as a primary oxidant was also studied. The results revealed an enhancement in the phenol removal due to the activation of PDS by α-FOD, and two different kinetics regimes were observed. First, fast phenol degradation was driven by a Fenton-like phenomenon within the first 30 min after the addition of PDS, in which the main reactive species were sulfate radicals. When all PDS is consumed, heterogeneous photocatalysis, almost negligible during the first phase, takes place due to the activity of α-FOD under visible light. During this second stage, and as already established for many heterogeneous photocatalysis reactions, hydroxyl radicals are the main reactive species.

In summary, the synthesized α-FOD demonstrated consistent performance across all tested operating conditions and exhibited several characteristics that suggest promising potential. Although further testing under natural or simulated solar light is still needed, the photocatalyst’s activity under visible-light irradiation—which accounts for approximately 47% of the solar spectrum, compared to only ~5% for UV—suggests that α-FOD may be a strong candidate for future solar-driven photocatalytic applications. Following this initial kinetics study, the next step to validate the relevance of this photocatalyst could be to design an optimized photoreactor to significantly reduce reaction times. On the other hand, the tests combining heterogeneous photocatalysis and a Fenton-like reaction with PDS as the primary oxidant demonstrated the excellent efficiency of the second mechanism for phenol degradation. This route needs to be explored further by testing other primary oxidants, such as H_2_O_2_ or PMS, as well as assessing the performances under simulated or natural sunlight, which could lead to an additional photo-Fenton-like effect.

## Figures and Tables

**Table 1:** Properties of the photocatalyst.

Parameter	Value
Crystalline phase	Monoclinic ferrous oxalate dihydrate (α-FOD)
Band gap (eV)	2.78 (direct transition) 2.28 (indirect transition)
d80 (µm)	18.9
Specific surface area (m^2^/g)	25.13
Total pore volume (cm^3^/g)	0.082
Average pore radius (nm)	6.5

**Table 2:** Comparison between α-FOD and other visible-active photocatalysts.

Photocatalyst	Dosage (g/L)	Phenol Initial Concentration (mg/L)	Removal (%)	Time (h)	Visible Light Source Power (Intensity)/Type	Reference
Sulfur and iron co-doped TiO_2_	1.0	20	99.4	10	1000 W (60 mW/cm^2^)/tungsten halogen lamp with UV cutoff filters)	[26]
Zinc-based MOF ([Cd_0.3_Zn_0.7_(oba)(4-bpdh)_0.5_]_n_·1.5DMF) ^1^	0.5	25	78.0	2	300 W (not reported)/xenon lamp	[27]
N-doped TiO_2_	0.2	100	6.6	5	100 W (38.5 mW/cm^2^)/not reported	[28]
LaVO_4_/MCM-48 ^2^	3.0	20	100	3	500 W (100 mW/cm^2^)/xenon lamp	[29]
α-FOD	1.0	10	98.2	9	18 W (49.3 mW/cm^2^)/LED lamp	This work

^1^ oba corresponds to oxybis benzoic acid, 4-bpdh refers to 4-diaza-2,4-hexadiene, and DMF to dimethylformamide. ^2^ MCM-48 corresponds to a mesoporous silica material.

## Data Availability

Dataset available on request from the authors.

## Supplementary Materials

The following supporting information can be downloaded at https://www.mdpi.com/article/10.3390/molecules30092059/s1, Figure S1: Scanning electron microscopies of the as-synthesized α-FOD at two different magnifications; Figure S2: Solid-state UV–Vis spectrum of the as-synthesized α-FOD; Figure S3: Point of zero charge of the α-FOD; Figure S4: Phenol concentration profiles for different photocatalyst dosages; Figure S5: Phenol removal experimental procedure flow chart.
