# Supplementary material for: Visible Light-Driven Phenol Degradation via Advanced Oxidation Processes with Ferrous Oxalate Obtained from Black Sands: A Kinetics Study
Source: Molecules. 2025 May 6;30(9):2059. doi: 10.3390/molecules30092059 (PMC12073611; doi:10.3390/molecules30092059)
Supplement: Supplementary file 1 [file molecules-30-02059-s001.zip › molecules-3531363-supplementary.pdf]

Supplementary material

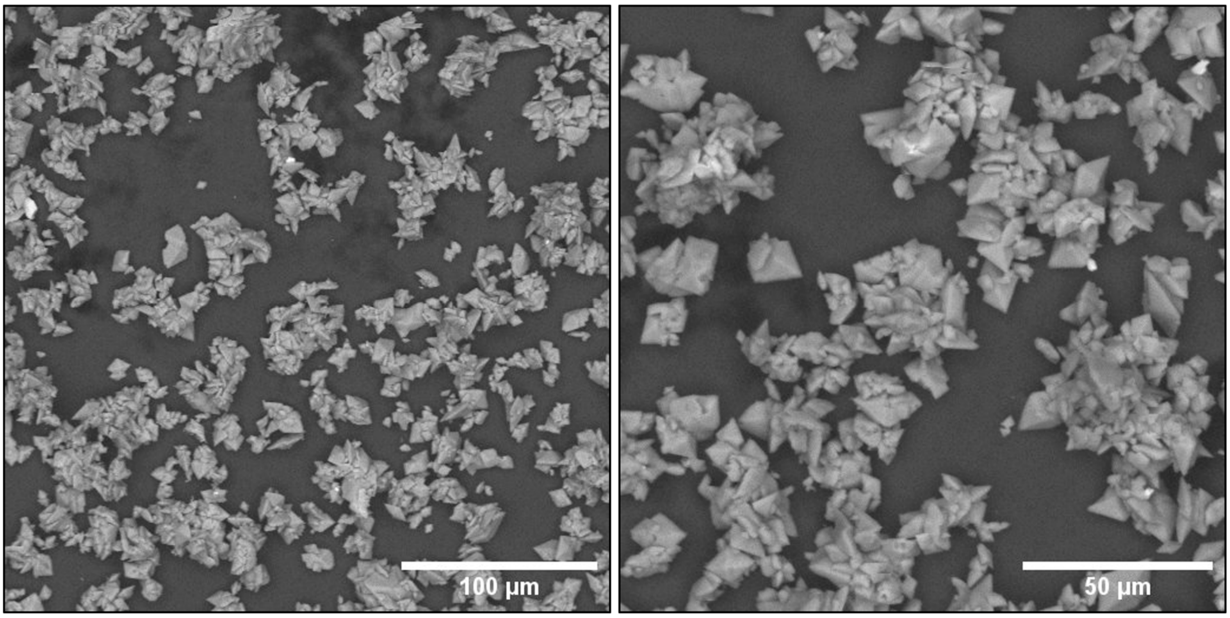

Figure S1. *Scanning electron microscopies of the as-synthesized α-FOD at two different magnifications*

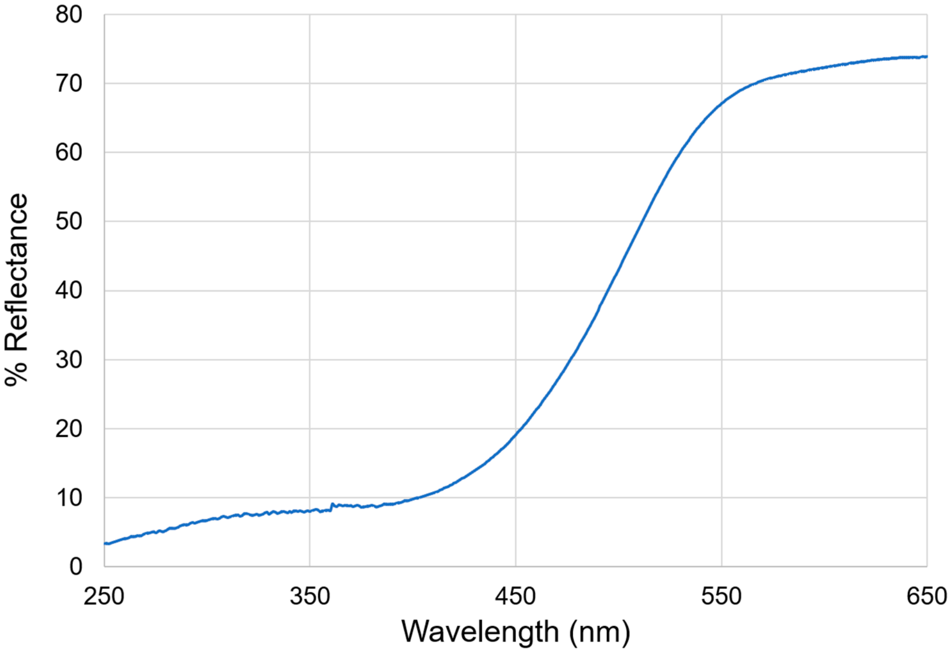

Figure S2. *Solid-state UV–vis spectrum of the as-synthesized α-FOD*

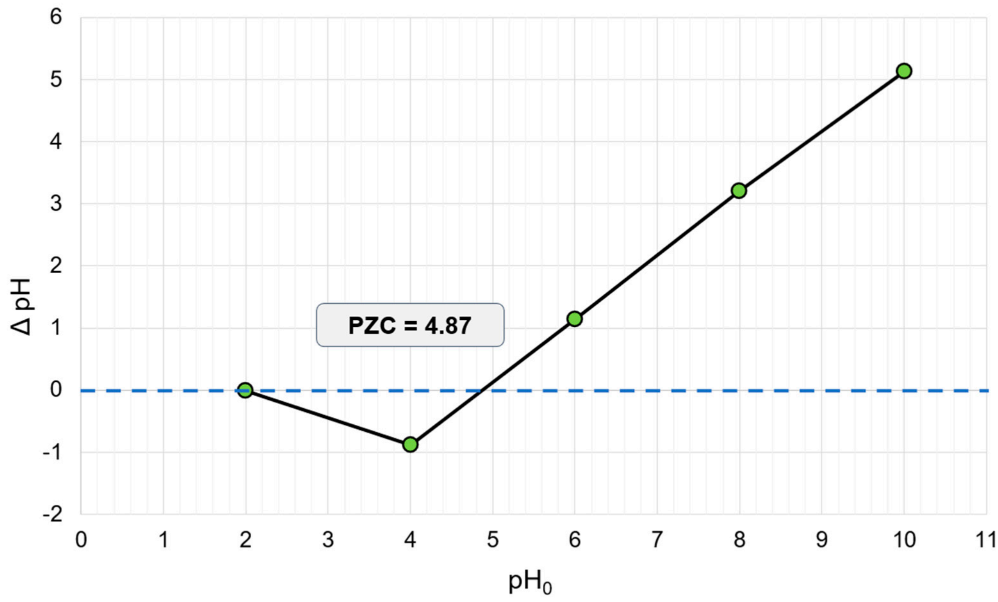

Figure S3. *Point of zero charge of the α-FOD*

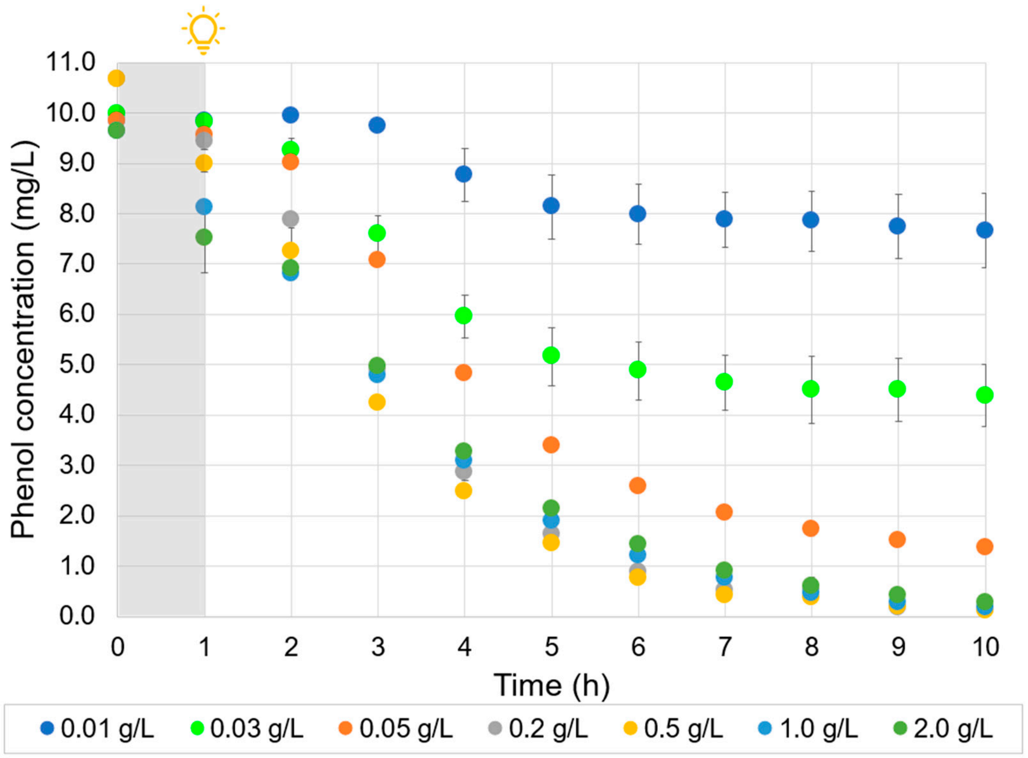

Figure S4. *Phenol concentration profiles for different photocatalyst dosages. Experimental conditions: Phenol initial concentration: ~10 mg/L, FOD dosage: 0.01 to 2.0 g/L, Experiment time: 1h under dark conditions + 9 h under visible light irradiation, neutral pH*

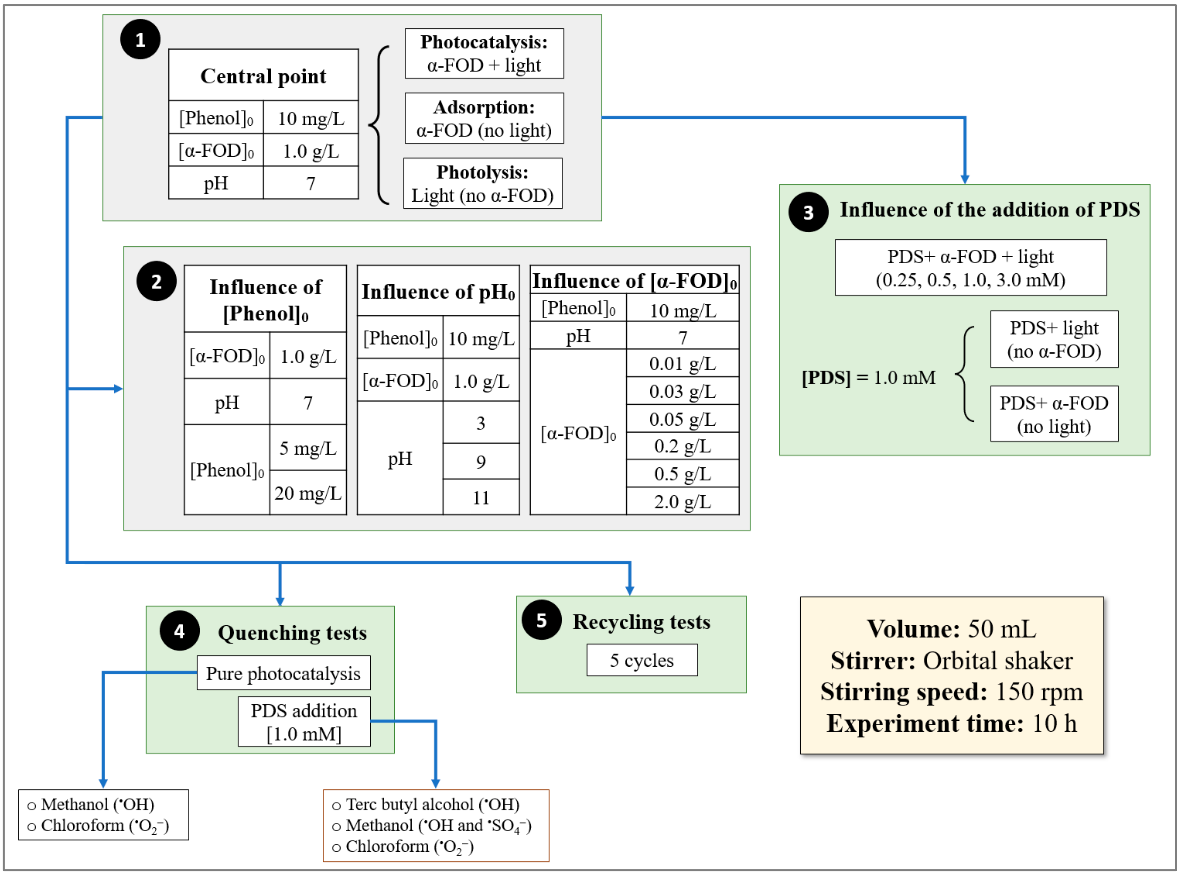

Figure S5. *Phenol removal experimental procedure flow chart*
